# Breaking Into Language in a New Modality: The Role of Input and Individual Differences in Recognising Signs

**DOI:** 10.3389/fpsyg.2022.895880

**Published:** 2022-05-18

**Authors:** Julia Elisabeth Hofweber, Lizzy Aumonier, Vikki Janke, Marianne Gullberg, Chloe Marshall

**Affiliations:** ^1^Department of Psychology and Human Development, Institute of Education, University College London, London, United Kingdom; ^2^School of Cultures and Languages, University of Kent, Canterbury, United Kingdom; ^3^Centre for Languages and Literature, Lund University, Lund, Sweden

**Keywords:** second language learning, iconicity, sign languages, implicit learning, first exposure, modality

## Abstract

A key challenge when learning language in naturalistic circumstances is to extract linguistic information from a continuous stream of speech. This study investigates the predictors of such implicit learning among adults exposed to a new language in a new modality (a sign language). Sign-naïve participants (*N* = 93; British English speakers) were shown a 4-min weather forecast in Swedish Sign Language. Subsequently, we tested their ability to recognise 22 target sign forms that had been viewed in the forecast, amongst 44 distractor signs that had not been viewed. The target items differed in their occurrence frequency in the forecast and in their degree of iconicity. The results revealed that both frequency and iconicity facilitated recognition of target signs cumulatively. The adult mechanism for language learning thus operates similarly on sign and spoken languages as regards frequency, but also exploits modality-salient properties, for example iconicity for sign languages. Individual differences in cognitive skills and language learning background did not predict recognition. The properties of the input thus influenced adults’ language learning abilities at first exposure more than individual differences.

## Introduction

Much language learning around the world takes place not in classroom settings involving explicit instruction but in contexts involving uninstructed, implicit learning. For example, many of us have travelled to countries where we do not speak the local language and have switched on the TV to watch the weather forecast in order to check if we will need an umbrella later that day. When confronted with novel input, the human brain cannot help but engage in implicit statistical learning processes (see Christiansen, 2019, for a discussion of this term). But how much can individuals learn about word forms in a new language from exposure to a short stretch of continuous language without training or instruction, and which language features and cognitive skills predict learning? These issues have received a lot of attention in second language acquisition research under different labels, such as incidental and implicit learning (DeKeyser, 2003; Hulstijn, 2003; Williams and Rebuschat, 2012), usage-based approaches (e.g., Ellis, 2012; Ellis and Wulff, 2020), statistical learning (e.g., Rebuschat and Williams, 2012; Christiansen, 2019) and artificial language learning (e.g., Saffran et al., 1997). This work has focused on spoken and written language. However, the problem of breaking down continuous linguistic input generalises to sign languages. Yet in sign languages much less is known about how this is achieved.

Here, we undertake the first study to investigate how adults who are naïve to sign languages break into a naturalistic stream of signs at first exposure. Specifically, we investigate whether sign-naïve viewers of a short video of naturalistic sign language can identify which sign forms they have and have not viewed and which features of the signed input and which cognitive skills are associated with successful identification. We aim to elucidate the features and skills that are common to learning across languages, regardless of modality, and those that might be particularly relevant to the learning of sign languages. In terms of the input, we zoom in on two factors predicted to influence sign language learning, i.e., frequency and iconicity.

## Background

### Input Processing

A pre-requisite for lexical acquisition is to identify word forms, and a key challenge for individuals who learn languages outside of classroom settings is to break down the continuous stream of naturalistic input to identify such strings. Since language does not come neatly segmented with words resembling ‘beads on a string’, this task requires learners to work on the input. This is one of the learner’s ‘problems of analysis’, as Klein (1986) puts it. A considerable body of research on spoken/written language has revealed that babies and adults alike appear to have sophisticated cognitive mechanisms for identifying word forms in a novel speech stream, which do not depend upon explicit instruction as to where word boundaries lie. Rather, a powerful statistical mechanism seems to keep track of frequency and transitional probabilities between adjacent and non-adjacent items to help identify patterns that translate into word forms and word boundaries, but also morphosyntactic and phonotactic patterns. For example, a range of studies has shown that child and adult learners are able to track the frequency of syllables and word forms for learning in both spoken (e.g., Saffran et al., 1996; Gomez and Gerken, 1999; Maye et al., 2002; De Diego Balaguer et al., 2007; Peters and Webb, 2018; Rodgers and Webb, 2020) and written contexts (e.g., Horst et al., 1998; Hulstijn, 2003; Waring and Takaki, 2003; Webb, 2005; Pigada and Schmitt, 2006; Pellicer-Sánchez, 2016). Generally speaking, higher frequency (both type and token) is associated with better learning. For example, Ellis et al. (2016) found that untutored learners of L2 English in the so-called ESF corpus (Perdue, 1993) acquired the most frequent and prototypical verbs in the input first (e.g., *put* and *give*), with a very high correlation between input frequency and learning. Moreover, type/token frequency and distributional properties have also been shown to interact with the salience of form, the importance of meaning, and the reliability of the form-meaning mappings (Ellis and Collins, 2009). Finally, the statistical capacity also operates on non-adjacent structures and situations. In a seminal paper, Yu and Smith (2007) showed that adults are able to track a particular word form across several situations when multiple possible referents are available, to ultimately determine the intended referent. This capacity for cross-situational learning also seems to scale up. Rebuschat et al. (2021) showed that adults are able to learn both vocabulary and grammar, words from different word classes, and in ambiguous contexts, which suggests a very powerful mechanism.

A great deal of research on input processing has drawn on the use of artificial languages, semi-artificial languages, or miniature languages, which provide researchers with total control over the distributional properties of the input to which learners are exposed (for useful overviews and discussions of these paradigms, see Hayakawa et al., 2020, for the lexicon; Grey, 2020, for morphosyntax; Morgan-Short, 2020, for neural underpinnings). While artificial languages have the advantage of allowing close experimental control over the properties of the input, their ecological validity has been questioned and in particular whether the properties of artificial and natural languages lead to the same learning outcomes and generalisations (e.g., Robinson, 2005, for a discussion). Nevertheless, much less work has been conducted on natural languages. A rare exception is a study by Kittleson et al. (2010) who tested implicit learning of Norwegian and showed that adults from different language backgrounds who were presented with continuous Norwegian speech in an implicit learning paradigm could segment the Norwegian speech stream and distinguish words from non-words after minimal exposure. Several studies have attempted to study the effects of input frequency as well as cognate status in classroom settings in which learners with different L1s were exposed to teachers of Polish with more or less control over actual input (Rast, 2008; Dimroth et al., 2013). Another series of experiments have attempted to emulate acquisition ‘in the wild’, or at least in a context replicating real-world context, while maintaining control over the input (Gullberg et al., 2010, 2012). In these studies, adults were exposed to 7 min of continuous and coherent speech in a language unknown to them, namely, Mandarin Chinese, in the form of a filmed weather forecast (Gullberg et al., 2010, 2012). Participants then undertook surprise tests of word recognition, word-meaning mapping, or phonological plausibility (as measured by lexical decision). The results suggested that adults exposed to naturalistic input in a novel language extracted information about this language without any additional explicit instructions (Gullberg et al., 2010) and that item frequency boosted word recognition, meaning mapping and phonotactic generalisation alike. Moreover, adults’ brains showed evidence of change in resting state connectivity as a function of such learning after only 14 min of exposure to continuous speech (Veroude et al., 2010).

#### Sign Languages

The problem of breaking down continuous linguistic input generalises to sign languages, yet in sign languages still less is known about how this is achieved, even in artificial language learning situations (exceptions are Orfanidou et al., 2010, 2015). The literature on spoken/written languages suggests that item frequency should matter for sign language too, but this prediction has not yet been tested.

Another important feature of sign languages is iconicity. Iconicity can be defined as a resemblance between a linguistic form and its meaning, where aspects of the form and meaning are related by perceptual and/or motor analogies (Sevcikova Sehyr and Emmorey, 2019). For example, in Swedish Sign Language, the sign for SNOW involves the open hands moving downwards as the fingers wiggle, resembling the movement of falling snowflakes. Although it has been argued that iconic mappings between form and meaning are more plentiful in speech than previously acknowledged (Perniss et al., 2010; Dingemanse et al., 2015), the visuo-gestural modality allows particularly rich opportunities for iconicity. It has also been argued that iconicity plays an important role for language learning. In spoken language acquisition, iconic manual gestures have been shown to boost L2 vocabulary acquisition in intervention studies, especially when learners repeat both spoken word form and gesture (see Gullberg, 2022, for an overview). In the case of sign language acquisition, the effects of iconicity on adult lexical acquisition are mixed (Ortega, 2017, provides a review) with positive effects on conceptual-semantic learning, but more mixed effects on form learning. It has also been suggested that in hearing learners of sign languages, the existing repertoire of iconic co-speech gestures may serve as a substrate for acquisition, facilitating form-meaning mappings in sign languages even at first exposure (Janke and Marshall, 2017; Ortega et al., 2019).

### Individual Cognitive Skills

Although all humans share the ability to acquire languages across the lifespan, research on second language acquisition of spoken languages suggests that individual differences affect the success of second language acquisition (Robinson, 2001; Paradis, 2011; Granena et al., 2016; Dörnyei and Ryan, 2015). For example, the influence of demographic factors, such as age on the ability to acquire another language, continues to be debated in the field (Birdsong, 2005; Singleton and Pfenninger, 2018). Moreover, cognitive abilities and executive functions, most notably phonological working memory, have been suggested to influence spoken language learning (O'Brien et al., 2007; Baddeley, 2017; Wen and Li, 2019). Another important factor affecting individuals’ ability to acquire another spoken language is their language aptitude, as measured by language learning aptitude tests (e.g., Meara, 2005; Artieda and Muñoz, 2016; Li, 2018). This raises the question of how variables that have been shown to modulate spoken second language acquisition operate when individuals acquire a new language in the visual modality.

To date, few studies have looked at the role of individual differences when learning sign languages. Existing studies of sign language learning under explicit conditions suggest that spoken vocabulary knowledge (Williams et al., 2017) and kinaesthetic and visuo-spatial short-term memory (Martinez and Singleton, 2018) predict learning of sign vocabulary, but that verbal short-term/working memory (Williams et al., 2017) and knowledge of other spoken languages (Martinez and Singleton, 2019) do not. However, the role of cognitive predictors in sign learning under implicit conditions at first exposure remains unstudied.

### The Current Study

In the current study, participants viewed 4 min of naturalistic, continuous sign language input in the form of a weather forecast presented in Swedish Sign Language (STS). Immediately after watching the forecast, they undertook a ‘surprise’ sign recognition task and judged whether or not individually presented signs had appeared in the forecast. Some of these signs had indeed appeared in the weather forecast (‘target signs’) but others had not (‘distractor signs’). We manipulated the frequency and iconicity of targets. With respect to the distractors, half were real signs of STS that had not appeared in the forecast but were phonologically similar to the targets (‘plausible distractors’), and half were *not* from STS: they were real signs of other languages, but they involved phonological features that are dispreferred (i.e., occur less frequently) across sign languages (‘implausible distractors’). Participants also completed a language background questionnaire and undertook a battery of tasks assessing their cognitive abilities (fluid intelligence, executive functions, visual attention, language learning aptitude, and L1 vocabulary knowledge; see the section ‘Materials and Procedures’. for detailed descriptions of the protocol). Our research questions and predictions were as follows:

1. Can sign-naïve adults successfully discriminate between signs that did appear in the forecast and signs that did not, and does doubling the exposure (to 8 min) increase performance accuracy?
  We predicted that although the task would be difficult, participants would distinguish between signs that they had viewed (‘target signs’) and signs that they had not viewed (‘distractor signs’). Furthermore, we predicted that performance accuracy would be enhanced by viewing the input twice compared to just once and that performance would be modulated by the input factors outlined in research questions 2 and 3, below.
2. Do frequency and iconicity impact how accurately target signs are recognised?
  We predicted that for target signs, those with greater occurrence frequency in the input would be recognised more accurately. We also predicted that target signs with greater iconicity would be recognised more accurately.
3. Does phonotactic plausibility impact how accurately distractor signs are identified?
  For distractor signs, we predicted that those that were phonologically implausible would be identified more accurately as not having been viewed in the input compared to signs that were phonologically plausible.
4. Which participant characteristics and cognitive skills are associated with greater recognition accuracy for target signs?
  Finally, we predicted that performance accuracy would be modulated by age, education, fluid intelligence, executive functions, visual attention, language learning aptitude, L1 vocabulary, and degree of multilingualism.

## Materials and Methods

### Participants

Our study was pre-registered on the Open Science Framework.^1^ In the pre-registration, we had indicated that we would test 100 participants, but data collection was suspended prematurely due to the onset of the COVID-19 crisis in spring 2020, resulting in a final sample size of 93. All participants were sign-naïve adults who were native speakers of English and resident in the United Kingdom. None had any known physical, sensory, or psychological impairments relevant to this study. Participants were randomly allocated to two Exposure groups: Exposure group 1x watched the weather forecast once (*N* = 50), Exposure group 2x watched the weather forecast twice back-to-back (*N* = 43). Their demographic and linguistic background was ascertained with a detailed questionnaire (see https://osf.io/ub28n/?view_only=fce4401c7284438d94d1ce52c7879733), administered immediately after the experiment using free online software (Surveymonkey, www.surveymonkey.co.uk). The general outline of our questionnaire was based on the Language History Questionnaire 2.0 (Li et al., 2014) but we created a bespoke set of questions tailored to our specific requirements. For instance, participants gave information on any prior exposure to sign languages, Makaton, fingerspelling or Swedish, because existing skills in these areas were exclusion criteria. We assessed education using two measures: (1) total number of years spent in formal education and (2) highest education level (1 = A-Level, 2 = Bachelor degree, 3 = post-graduate degree, and 4 = doctoral degree). Participants were aged between 18 and 40. The upper age limit was applied due to the reported detrimental effects of Age on some of our key variables, in particular on visual search abilities (Hommel et al., 2004). Although we aimed for a comparable gender split between groups, our groups could not be gender-matched due to the interruption of data collection in spring 2020: Exposure group 1x has marginally more females (females: 84%, *N* = 42, males: 16%, *N* = 8) than Exposure group 2x (females: 63%, *N* = 27, males: 37%, *N* = 16) [Chi-squared (1,93) = 5.34, *p* = 0.05].

Given that individuals’ language background impacts upon their ability to benefit from naturalistic input (Ristin-Kaufmann and Gullberg, 2014), we assessed participants’ language history and usage. Our data set comprised both monolinguals and multilinguals, but we kept language dominance profiles constant: all participants were native speakers of English and reported English as their most commonly used language. However, we predicted that variability in the degree of multilingualism would affect performance on the sign recognition task, so in our measures, components of multilingualism were classed as continuous, rather than categorical, to do justice to the high levels of individual variability that characterise the phenomenon of multilingualism (Luk and Bialystok, 2013). For each of their languages, participants reported Age of Onset, the current frequency of usage (six-point Likert scale), and the extent to which their languages had been acquired through explicit vs. implicit learning (six-point Likert scale, ranging from ‘mostly formal’ to ‘mostly informal’). This information generated the following set of predictors for our regression analyses: number of languages learnt, number of additional languages, multilingual usage scores (sum of frequency scores reported for each language), number of languages acquired in an informal context that is through implicit learning. Finally, participants were asked to report the frequency with which they engaged in code-switching between languages (six-point Likert scale) as this may modulate executive functions, which in turn benefit language learning (Hofweber et al., 2020).

Table 1 presents descriptive statistics for the demographic and linguistic variables from the questionnaire and inferential statistics from a multivariate ANOVA with the between-group variable Exposure group (exposure 1x vs. exposure 2x) and the various background variables as dependent variables. This revealed that the two groups did not differ on any background variables, although the group difference in Age approached significance [*F*(1,92) = 3.96, *p* = 0.05, *η^2^* = 0.04]. Moreover, there was a slight trend for Exposure 1x group to display greater levels of multilingualism than Exposure 2x group, as evidenced by a greater number of languages overall and of additional languages, but these differences did not reach significance. All participants completed a test battery assessing their cognitive abilities, such as executive functions, language aptitude, L1 vocabulary knowledge and fluid intelligence (see section 2.2.3. for details). Table 2 presents a comparison of cognitive background measures for each exposure group. A multivariate ANOVA with Exposure group (1x, 2x) as the between-subject variable revealed that the 1x Exposure group performed better at Kinaesthetic working memory and Llama D, but displayed less good visual search abilities. Thus, the background measures suggest that the two Exposure groups were matched on the most crucial background variables, such as Age, Fluid Intelligence, and executive functions, but differed slightly on Kinaesthetic working memory, Llama D, and visual search abilities.

**Table 1:** Demographic and linguistic background variables by exposure group.

Variables	Group	*Mean*	*SD*	*F*	*p*	*η^2^*
Age (years)	Exposure 1x	25.56	6.38	3.96	0.05	0.04
	Exposure 2x	28.19	6.30			
Education (years)	Exposure 1x	17.20	2.72	1.05	0.31	0.01
	Exposure 2x	17.81	3.06			
Education (level)	Exposure 1x	2.60	0.76	0.07	0.80	0.00
	Exposure 2x	2.56	0.83			
Languages (number)	Exposure 1x	4.02	1.24	3.26	0.07	0.03
	Exposure 2x	3.60	0.93			
Non-native languages (number)	Exposure 1x	2.76	1.27	3.26	0.06	0.04
	Exposure 2x	2.30	1.01			
Multilingual usage score (sum of frequencies)	Exposure 1x	12.50	3.83	0.17	0.68	0.00
	Exposure 2x	12.19	3.34			
Informally acquired languages	Exposure 1x	2.10	0.96	0.72	0.40	0.01
(number)	Exposure 2x	2.28	1.03			
Code-switching frequency (1–6)	Exposure 1x	2.26	1.40	1.87	0.18	0.02
	Exposure 2x	1.84	1.59			

**Table 2:** Cognitive background variables by exposure group.

Variables	Group	*Mean*	*SD*	*F*	*p*	*η^2^*
Fluid intelligence (WAIS matrices)	Exposure 1x	21.58	2.43	0.01	0.93	0.00
	Exposure 2x	21.63	2.79			
Inhibitory control (flanker effect)	Exposure 1x	61.03	17.76	0.01	0.93	0.00
	Exposure 2x	60.69	20.17			
Phonological working memory (digit span)	Exposure 1x	10.76	2.10	0.04	0.85	0.00
	Exposure 2x	10.67	2.26			
Visuo-spatial working memory (Corsi span)	Exposure 1x	6.14	1.26	1.53	0.22	0.02
	Exposure 2x	5.84	1.07			
Kinaesthetic working memory	Exposure 1x	10.92	1.59	4.75	0.03	0.05
	Exposure 2x	10.06	2.19			
Visual search load effect	Exposure 1x	254.78	159.54	11.69	0.001	0.11
	Exposure 2x	164.43	72.68			
Llama B (language aptitude)	Exposure 1x	57.80	18.16	1.55	0.22	0.02
	Exposure 2x	53.02	18.81			
Llama D (language aptitude)	Exposure 1x	29.80	13.01	4.091	0.046	0.04
	Exposure 2x	23.95	14.86			
L1 vocabulary (WAIS)	Exposure 1x	38.74	5.98	0.011	0.92	0.00
	Exposure 2x	38.88	7.43			

### Materials and Procedures

Our experimental protocol was approved by the first and last authors’ institutional review board and was carried out in accordance with the provisions of the World Medical Association Declaration of Helsinki. All tasks were administered in the same session on the same day. The overall duration of the experimental protocol was 1.5 h. The protocol followed a blended approach combining fixed and counterbalanced administration orders. To avoid priming from other tasks, participants first conducted the implicit learning task, i.e., the weather forecast in Swedish Sign Language. They were not aware that they would be tested on the weather forecast content afterwards. Immediately after viewing the forecast, participants undertook a ‘surprise’ sign recognition task, in which they indicated whether or not they recognised signs from the forecast. Following the administration of the weather forecast materials, participants completed the online background questionnaire. After that, a battery of individual differences tasks was administered: five executive function tasks (administered in a partially counterbalanced order), three verbal tasks, and a task assessing fluid intelligence. All tasks were administered face-to-face on an individual basis in a lab setting using a Dell XPS 13 Laptop with a 13-inch screen. The following sub-sections describe the materials and procedures. All materials related to the STS weather forecast, sign recognition task and iconicity rating task are available at https://osf.io/ub28n/?view_only=fce4401c7284438d94d1ce52c7879733.

#### Naturalistic Input: Weather Forecast in Swedish Sign Language

The weather forecast is a particular discourse type aimed at the general public and likely to be familiar to most people. It functions within a fairly rigid framework, whereby listeners/viewers have expectations about the sorts of words (e.g., weather types, temperatures, geographical locations, and times of the day/days of the week), images (e.g., a map of a country overlain with weather symbols), and gestures (e.g., points to areas of the map) that will occur (Moore Mauroux, 2016). This discourse type was chosen not only because it was used in previous first-exposure studies of spoken language (Gullberg et al., 2010, 2012), but also because it could be adapted for presentation in Swedish Sign Language and still retain its familiarity for viewers.

Few examples of weather forecasts delivered in sign languages exist. Most are interpretations into sign language of a spoken language forecast, whereby the signing interpreter is not directly in front of the weather map but is to the edge of the screen and it is the speaking forecaster who is interacting directly with the map. We required a forecast in which the forecaster interacts directly with the map and wanted to maintain experimental control of sign frequency, so we created a weather forecast specifically for this project. The script was originally written in English, then translated into Swedish and then interpreted by a professional interpreter from Swedish into Swedish Sign Language (STS). The aim was to create as natural, engaging and professional-looking a forecast as possible given our constraints. By using STS as a target language, we avoided a sign language where the mouthings could be related to the sound patterns of English words: we did not want English participants to extract information about signs’ meanings from the signer’s lip movements.

Our weather forecast video lasted 4 min and was constructed around 22 target signs that covered a variety of semantic meanings relevant to a weather forecast, including weather-related words (e.g., *rain*, *sun*, and *cloud*), temperature-related words (e.g., *warm*, *cold*, and particular numbers), geography-related words (e.g., *north*, *south*, and *mountain*), and time-related words (*today*, *night*). An important experimental manipulation was that the 22 target signs varied in their occurrence frequency. Eleven of them occurred eight times in the forecast, whilst the other eleven occurred three times [there was one exception: the item ‘söder’ (south) appeared four times instead of three times; the additional token was introduced by mistake during the translation stage from English to Swedish]. The former set was therefore designated ‘high frequency’ signs, the latter ‘low frequency’ signs. Both sets were matched for aspects of sign language phonology, namely for locations of signs and hand configurations and for the number of one-handed signs vs. two-handed signs where both hands move, vs. two-handed signs where the active hand contacts a static non-dominant hand.

The target signs were also matched for iconicity, with both sets containing items that ranged from low to high iconicity on the basis of ratings from an independent group of 24 British English-speaking sign-naïve raters. Iconicity of the target items was assessed using an iconicity rating task based on Motamedi et al. (2019). Participants saw each target sign and its translation individually on a PowerPoint slide and rated the iconicity of each sign on a scale from 1 (not iconic) to 7 (very iconic). The ratings showed that the high (*M* = 3.64, *SD* = 1.55) and low frequency (*M* = 3.68, *SD* = 1.76) signs did not differ in their level of iconicity [*F*(1,22) = 0.003, *p* = 0.96, *η*^2^ = 0.000].

The iconicity ratings for each target sign are provided in the supplementary materials.^2^ An example of a sign rated highly iconic is the sign for ZERO, in which the fingers form a circle. In contrast, an example of a low iconicity sign is the sign for WARM, which is represented by the signer’s hand brushing past their chin. Short videos displaying each target sign can be viewed in the supplementary materials: https://osf.io/kf2nr/.

#### Sign Recognition Task

The sign recognition task was programmed and administered using PsychoPy 1.85. Its administration took approximately 5 min. Participants viewed 66 short videos of individual signs and indicated by key press whether they had viewed a given sign in the forecast or not. If they thought they had seen the sign, they pressed the ‘Yes’ key, marked by a sticker on the left arrow button of the keyboard. If they thought they had not seen the sign, they pressed the ‘No’ key, marked by a sticker on the right arrow button of the keyboard. Signs were presented without any accompanying mouthing and were chosen to generate three different item conditions:

1. *Target items (N = 22)*: signs of STS that had occurred in the weather forecast;
2. *Plausible distractors (N = 22)*: signs of STS that had not occurred in the forecast but were phonologically similar to the target signs; and
3. *Implausible distractors (N = 22)*: signs that had not occurred in the forecast and were not signs of STS. Although they were real signs from other sign languages (in order to ensure ecological validity), they included phonological features that—to the extent of our current knowledge of sign formation—are dispreferred (and therefore rare) within lexical signs across the world’s sign languages (Sandler, 2012). This is because they break the formational constraints of selected fingers (for one-handed signs) or the dominance/symmetry constraints (for two-handed signs). As a result, we predicted that participants would not confuse them as readily with the target signs, so would reject them more accurately.

The correct response for Target items was ‘Yes’, whilst for Plausible and Implausible Distractors it was ‘No’. Recall that the target items were further subdivided by their frequency of occurrence in the weather forecast, that is *high frequency items* occurring 8x in the forecast (*N* = 11) and *low frequency items* occurring 3x in the weather forecast (*N* = 11). In addition, target signs were categorised by iconicity, as detailed above. Items with scores above 3.5 were classified as *high iconicity items* (*N* = 11); those with scores of 3.5 or below were considered *low iconicity items* (*N* = 11). The combination of the frequency and iconicity criteria resulted in six high iconicity–high frequency items, six low iconicity–low frequency, five high iconicity–low frequency and five low iconicity–high frequency items.

**Figure 1:**
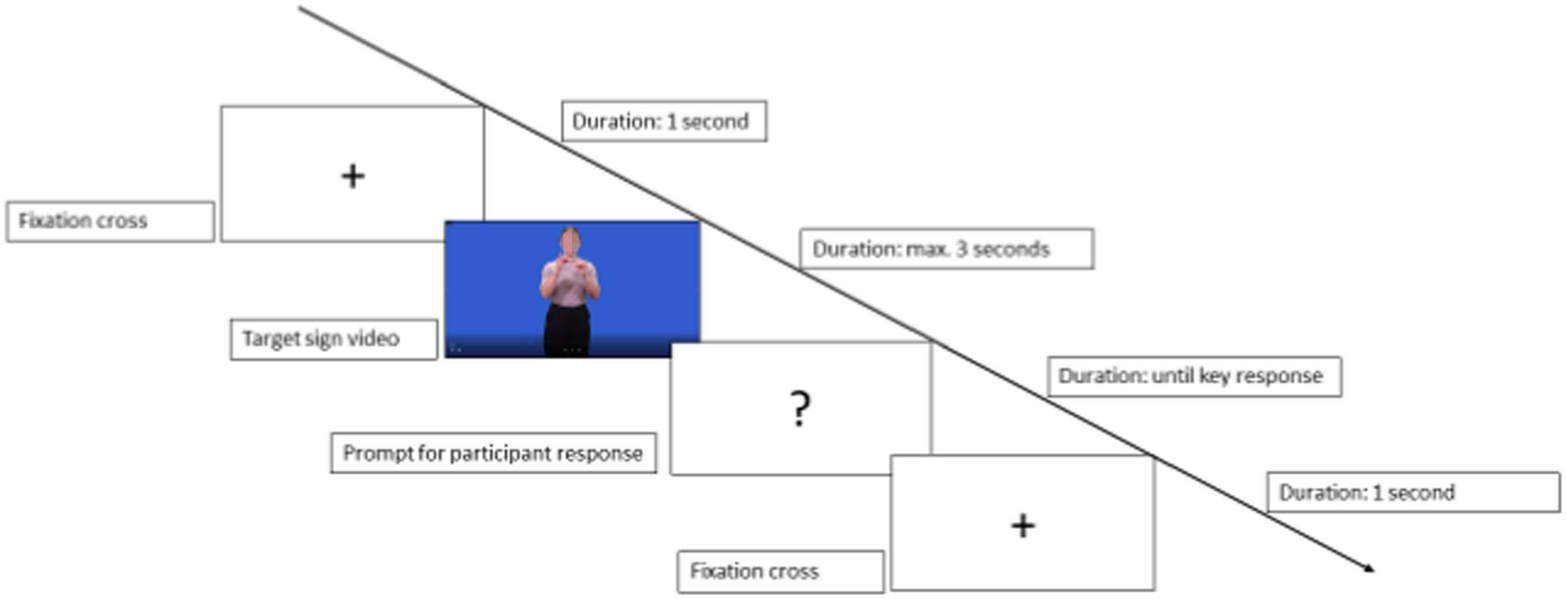
Structure of a trial in the sign recognition task.

The experimental task was preceded by four practice trials, after which the instructions were repeated and the first trial began. Figure 1 summarises the structure of a trial. Participants saw a fixation cross on the screen for 1 s, followed by a stimulus video, the duration of which varied but never exceeded 3 s. After the stimulus video, a question mark appeared on the screen, prompting yes–no responses. Response times were measured from the onset of the video and were not capped, although participants had been instructed to respond as fast as possible to encourage intuitive reactions. Once they had responded, they were taken to the next trial. All items were presented in a different fully randomised order for each participant.

#### Individual Differences Battery of Cognitive Tasks

We administered a battery of tasks assessing individual differences which have been implicated in adult language learning, such as cognitive abilities [general executive functions (inhibitory control, phonological working memory), language aptitude, vocabulary size in the first language, and fluid intelligence]. We also assessed executive functions that we expected to impact on sign language learning, namely, visual search abilities, visuo-spatial working memory, and kinaesthetic working memory. All tasks were designed with the aim of generating continuous predictor variables suitable for use in linear regression models. The administration duration of each task was approximately 5 min.

#### Tasks Assessing Executive Functions

##### Flanker Task

The Flanker task was based on the high-monitoring version of Eriksen and Eriksen’s (1974) Flanker task, as described by Costa et al. (2009), and was created using PsychoPy version 1.8. This task assessed inhibitory control by comparing performance in trials requiring inhibitory control to performance in baseline trials. Participants saw a row of five arrows, presented horizontally. They had to indicate the direction of the central arrow by pressing the left arrow key for a left-facing central arrow and the right arrow key for a right-facing central arrow. In congruent trials, all arrows face the same direction, so no inhibition is required. In incongruent trials, the central target arrow faces a different direction to its surrounding four arrows. To succeed on the task, participants must use inhibitory control to suppress the distractor arrows. In our version of the task, the congruent–incongruent trials were evenly split (48 congruent vs. 48 incongruent). Inhibitory control is measured as the performance difference between incongruent and congruent trials. The task is available at https://osf.io/ub28n/?view_only=fce4401c7284438d94d1ce52c7879733.

##### Visual Search Task

This task was sourced from the open-access Psytoolkit website https://www.psytoolkit.org/. It assessed individuals’ ability to identify a specified target under different conditions of visual search load. Participants saw a display of four versions of the capital letter ‘T’ (blue T, orange T, upside-down blue T, and upside-down orange T) and were instructed to press the space bar once they had identified the target T, which was defined as the non-inverted orange T. Trials without the target did not require a response. Overall, the task comprised 50 individual trials that differed in visual search load as a function of the number of distractor stimuli present in the display, that is 5, 10, 15, or 20 distractor stimuli. Visual search performance was calculated by comparing RTs in the high load conditions (20 and 15 distractors) with those in the low load conditions (5 and 10 distractors).

##### Visuo-Spatial Working Memory Task

Visuo-spatial working memory was assessed using the *Corsi forward span*, sourced from the open-access Psytoolkit website https://www.psytoolkit.org/. Participants saw nine pink squares on the laptop screen. In each trial, some of the pink squares light up in yellow in a certain order, and participants are instructed to click on the blocks that have lit up in the order that was shown. The number of blocks gradually rises, increasing the load on visuo-spatial working memory. Participants’ Corsi span score is the highest number of squares they memorise at least twice in a row.

##### Phonological Working Memory Task

We used the version of the *digit forward span* created for the WAIS III test battery (Wechsler, 1997; proprietary material that cannot be shared). Participants listened to pre-recorded sequences of digits, which they had to repeat. The number of digits gradually increased. Participants’ phonological working memory score was the raw score of correct responses.

##### Kinaesthetic Working Memory Task

The design and materials of this task were based on Wu and Coulson (2014), retrieved from https://bclab.ucsd.edu/movementSpanMaterials/. Participants watched short 3-s videos of a series of individual hand and arm movements and were instructed to repeat the movements in the same order. Their replications of the movements were video-recorded. At each span level, the number of movements increased, with each span level comprising two trials. Whilst Wu and Coulson’s (2014) task progressed to span level 5, we stopped at span level 3 because piloting had revealed floor effects beyond this span. Participants’ kinaesthetic working memory score was calculated as their raw number of correct responses. When scoring the task, we followed the guidelines provided by Wu and Coulson (2014). Results from a subset of 12 randomly selected participants were scored by two independent judges (first and last authors), whose scores converged highly [*r*(1,12) = 0.90, *p* < 0.001].

#### Tasks Assessing Linguistic Skills and Fluid Intelligence

##### Vocabulary Size in the First Language

We administered the English vocabulary test of the Wechsler Adult Intelligence Scale WAIS IV (Wechsler et al., 2008; proprietary material that cannot be shared). Participants were presented with 26 English lexical items and asked to provide a definition for each item. Items were presented aurally and visually using PowerPoint slides. Responses were recorded using Audacity and subsequently transcribed and scored based on the detailed WAIS IV scoring manual. To ascertain that the scoring was reliable, the data from a subset of 20 randomly selected participants were scored by two independent judges (first and last authors). This process resulted in an interrater correlation score of *r* = 0.94 at a significance level of *p* < 0.001.

##### Language Learning Aptitude Tests

To assess general language aptitude, we administered the Llama B and D sub-sections of the Llama tests (Meara, 2005, as sourced from the Lognostics website in August 2019, https://www.lognostics.co.uk/tools/llama/). LLAMA test scores have been found to correlate with scores in grammaticality judgment tests (Abrahamsson and Hyltenstam, 2008), morphosyntactic attainment (Granena, 2012), collocation knowledge (Granena and Long, 2013), and pronunciation (Granena and Long, 2013). The Llama B test assessed vocabulary learning skills. Participants were presented with 20 images of imaginary animals on the laptop screen. Each animal had a name, which could be revealed by clicking on its screen image. The task consisted in learning as many of the name–stimulus associations as possible within a given time frame of 2 min. Subsequent to this learning phase, participants were tested on their knowledge of the animal names. The Llama D test tapped into implicit phonological language learning. Participants listened to words presented as strings of sound sequences. Subsequently, they were presented with words aurally and asked to make a judgment as to whether or not they had just heard the word. Participants received points for correct responses, but were penalised for incorrect ones.

##### Fluid Intelligence

Participants’ pattern recognition and logical reasoning ability was assessed using the Matrices component of the Wechsler Adult Intelligence Scale WAIS III (Wechsler, 1997; proprietary material that cannot be shared). This task was completed using pen and pencil. They were presented with sequences of shapes and colours. Each sequence contained a gap. At the bottom of the page, participants encountered five possible shapes that were potential solutions to fill the gap in the sequence. They were asked to select the shape that should logically be used to fill that gap. We used the raw scores based on the total number of correct responses as an indicator of fluid intelligence.

### Analyses

The aim of this study was to investigate the predictors of successful sign recognition on first exposure to minimal input.

The first analysis assessed participants’ performance in the different item conditions (targets, plausible distractors, and implausible distractors), thus addressing research questions 1 and 3. We also investigated whether the influence of input factors interacted with the number of times participants had been exposed to the weather forecast, that is the between-subject factor Exposure Group (1x, 2x).

The second analysis focused on the properties of target items, that is research question 2. To investigate the impact of the characteristics of the input materials, the following variables were entered into the mixed models: Frequency of target items (high vs. low) and Iconicity of target items (a continuous variable with a rating scale from 1 = low to 7 = high).

The third analysis explored research question 4, which focused on predictors of accuracy in terms of individual differences between participants.

In all analyses, we used the lme4 and lmer.test package in *R*, which allows for the use of mixed models and automatically provides the results of significance testing in the form of a value of *p* (Kuznetsova et al., 2017). Binary variables were centred using sum-coding by assigning the values −1 and +1, as suggested by Winter (2019). An exception was the analyses comparing accuracy to chance; in these analyses we used the non-centred versions of the fixed effect variables. When taking random effects into consideration, we assumed a maximally conservative approach, allowing both items and subjects to vary by both intercept and slope.

## Results

### Performance Across Item Conditions

The sign recognition task generated a total of 6,138 data points across 93 participants and 66 items. All data points were included in the analyses, except for responses with Reaction times below 150 ms, which were excluded based on the assumption that they represented slips of the finger or premature guesses. Table 3 displays the average Accuracy rates (Number of correct trials/Number of total trials) for each experimental condition.

**Table 3:** Accuracy rates by condition.

Accuracy rates in % condition	Number of participants	Mean	(*SD*)	Minimum	Maximum
Targets	93	53	(14)	23	86
Plausible distractors	93	60	(14)	23	95
Implausible distractors	93	64	(15)	27	95

To establish differences in accuracy across conditions and how these may have interacted with the number of times participants had viewed the forecast, we created a mixed model using the glmer function (family = ‘binomial’) with Accuracy (accurate, inaccurate) as the dependent variable and Condition (targets, plausible distractors, and implausible distractors) and Exposure group (1x, 2x) as the predictors. Table 4 summarises the model output.

**Table 4:** Model output of glmer for accuracy by condition and group.

Random effects	Variance		*SD*	
Subject	0.05		0.21	
Item	0.61		0.78	
Fixed effects	*B*	*SE*	*Z*	*p*
Intercept (targets)	0.16	0.18	0.93	0.36
Plausible distractors	0.27	0.25	1.09	0.27
Implausible distractors	0.50	0.25	2.00	0.045
Group	0.06	0.05	1.17	0.24
Plausible distractors: group	−0.11	0.07	−1.55	0.12
Implausible distractors: group	−0.12	0.07	−1.80	0.07

Glmer (accuracy ~ condition*group + (1 + 1|subject) + (1 + 1|item), data, family = ‘binomial’).

**Figure 2:**
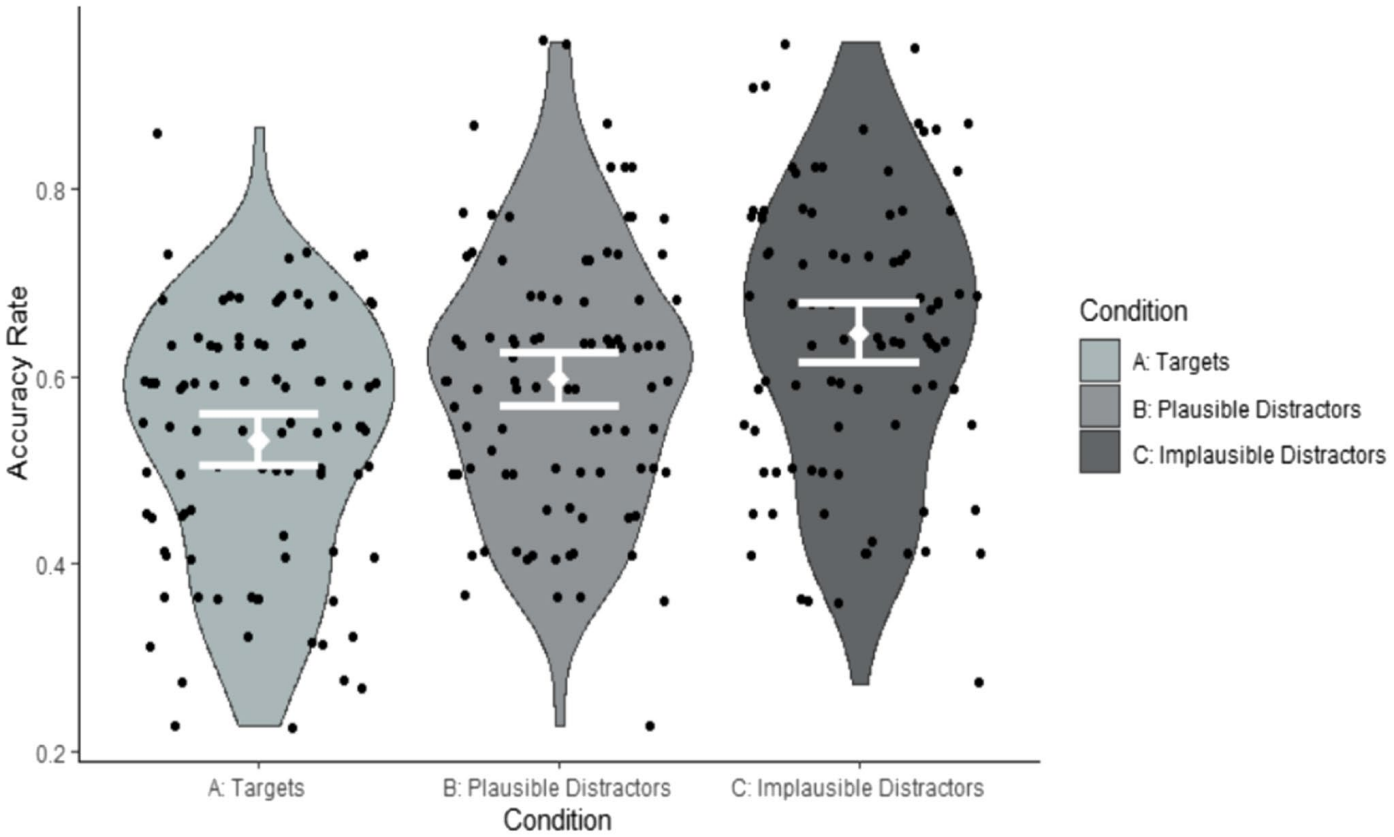
Accuracy rates by condition summarised by participants (Correct response Condition A: Yes; Correct response Conditions B and C: No).

**Figure 3:**
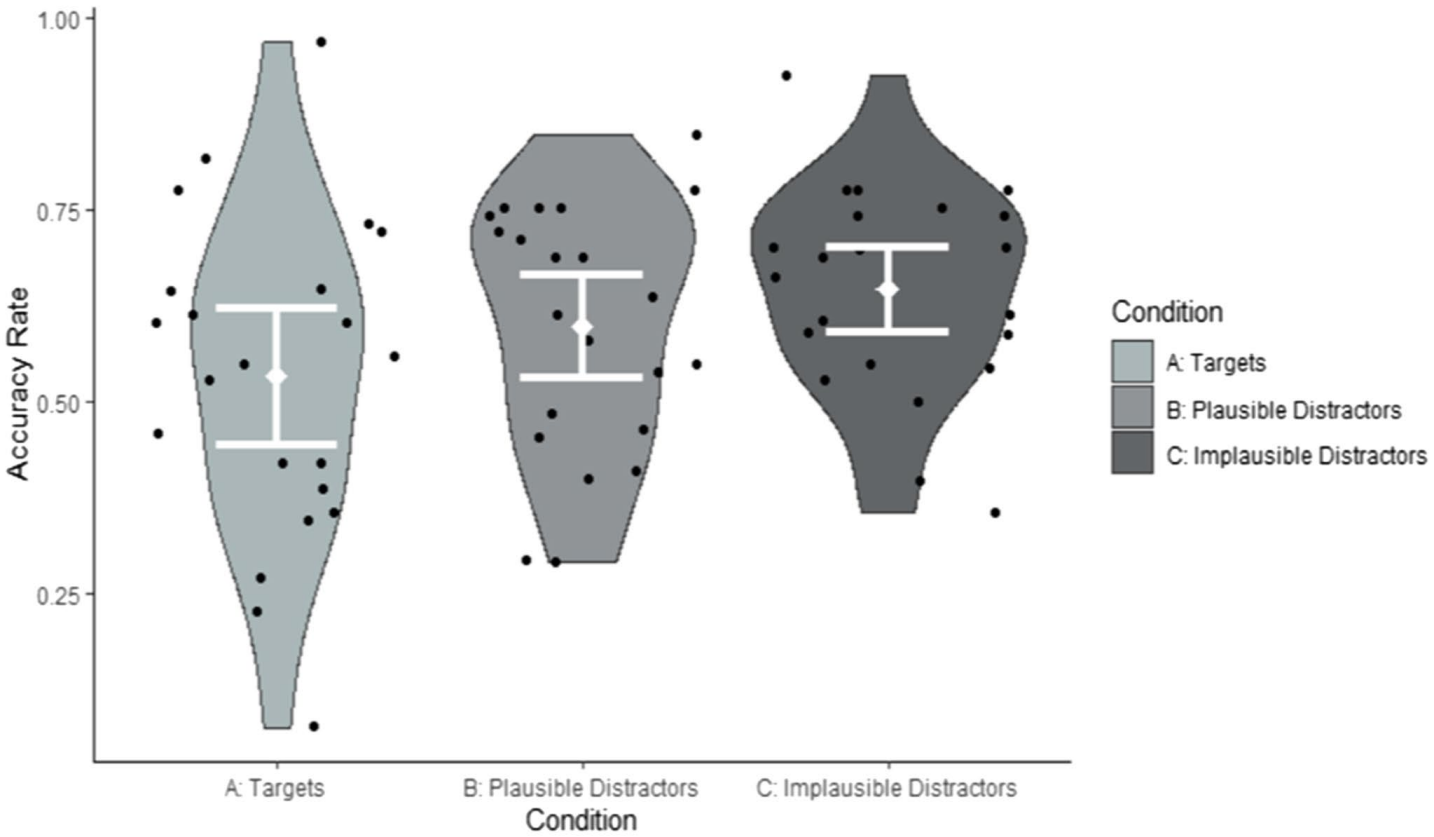
Accuracy rates by condition summarised by items (Correct response Condition A: Yes; Correct response Conditions B and C: No).

As can be seen from Table 4, the only significant effect was the variable ‘implausible distractors’. However, the *post-hoc* pairwise comparisons using the *emmeans* function in *R* (Winter, 2019) did not reveal any significant differences in accuracy between conditions. All pairwise comparisons were associated with *p* values in excess of 0.2. Crucially, the effect of Exposure group was not significant and accuracy across the three conditions did not interact with Exposure group. Participants who had viewed the weather forecast twice were not more accurate than those who had viewed it once. Hence, Exposure group was not included in our further accuracy analyses. Figures 2, 3 illustrate these findings by participant and by item.

We subsequently compared recognition performance in the three conditions to chance by constructing a mixed glmer model (family = ‘binomial’) from which the intercept was removed and the fixed factor Condition was entered in its non-centred version. The dependent variable was Accuracy (accurate, inaccurate), and the predictor variable was Condition (targets, plausible distractors, and implausible distractors). Table 5 presents the random and fixed effects.

**Table 5:** Model output for the comparison of accuracy to chance by condition.

Random effects	Variance		*SD*	
Subject	0.046		0.21	
Item	0.61		0.78	
Fixed effects to chance	*B*	*SE*	*Z*	*p*
Targets	0.16	0.18	0.90	0.37
Plausible distractors	0.43	0.17	2.49	0.01
Implausible distractors	0.66	0.17	3.77	0.0002

Glmer (accuracy ~ −1 + condition + (1 + 1|subject) + (1 + 1|item), data, family = ‘binomial’).

As can be seen from Table 5, participants performed at chance on target items. However, on plausible and implausible distractor items they performed significantly above chance. The size of the effect of above-chance performance was greater for the implausible than for the plausible items [Targets: *Cohen’s D* = 0.09; Plausible items: *Cohen’s D* = 0.26; Implausible items: *Cohen’s D* = 0.40, where *Cohen’s D* = B/(SQRT(N)*SE)], suggesting that accuracy was greater in the implausible than in the plausible condition. Importantly, a large proportion of variance was explained by random effects due to items (variance = 0.6086). Figures 2, 3 suggest that this item variability was greatest in the target condition. To explore the effects of items in greater detail, we investigated the impact of iconicity and frequency, which we had predicted would modulate accuracy in the target condition. As can be seen from the random effects, the variance associated with differences between individual participants was only small (variance = 0.0456).

### The Effects of Input Factors on Target Item Recognition

**Figure 4:**
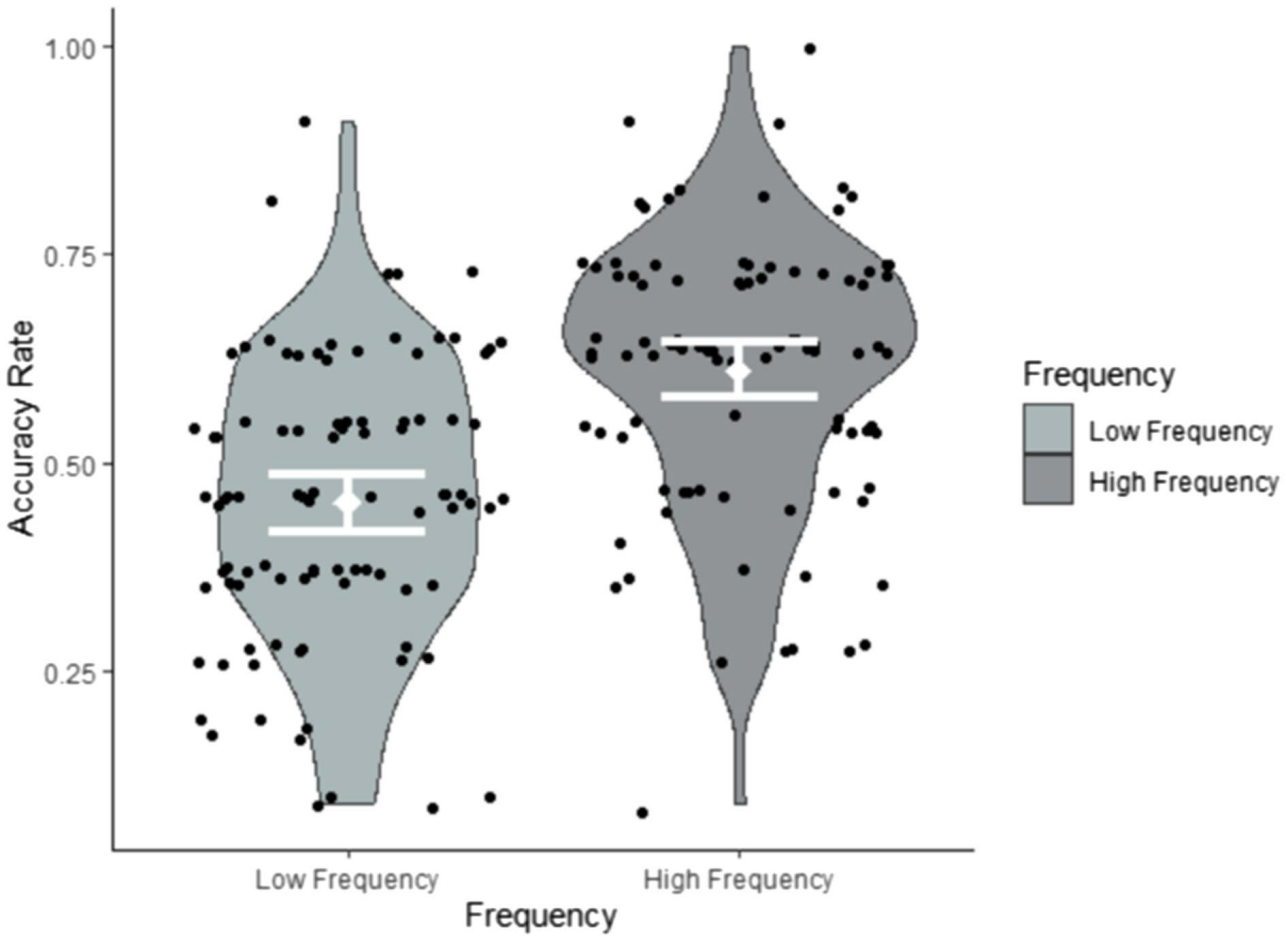
Accuracy rates by frequency summarised by participants.

**Figure 5:**
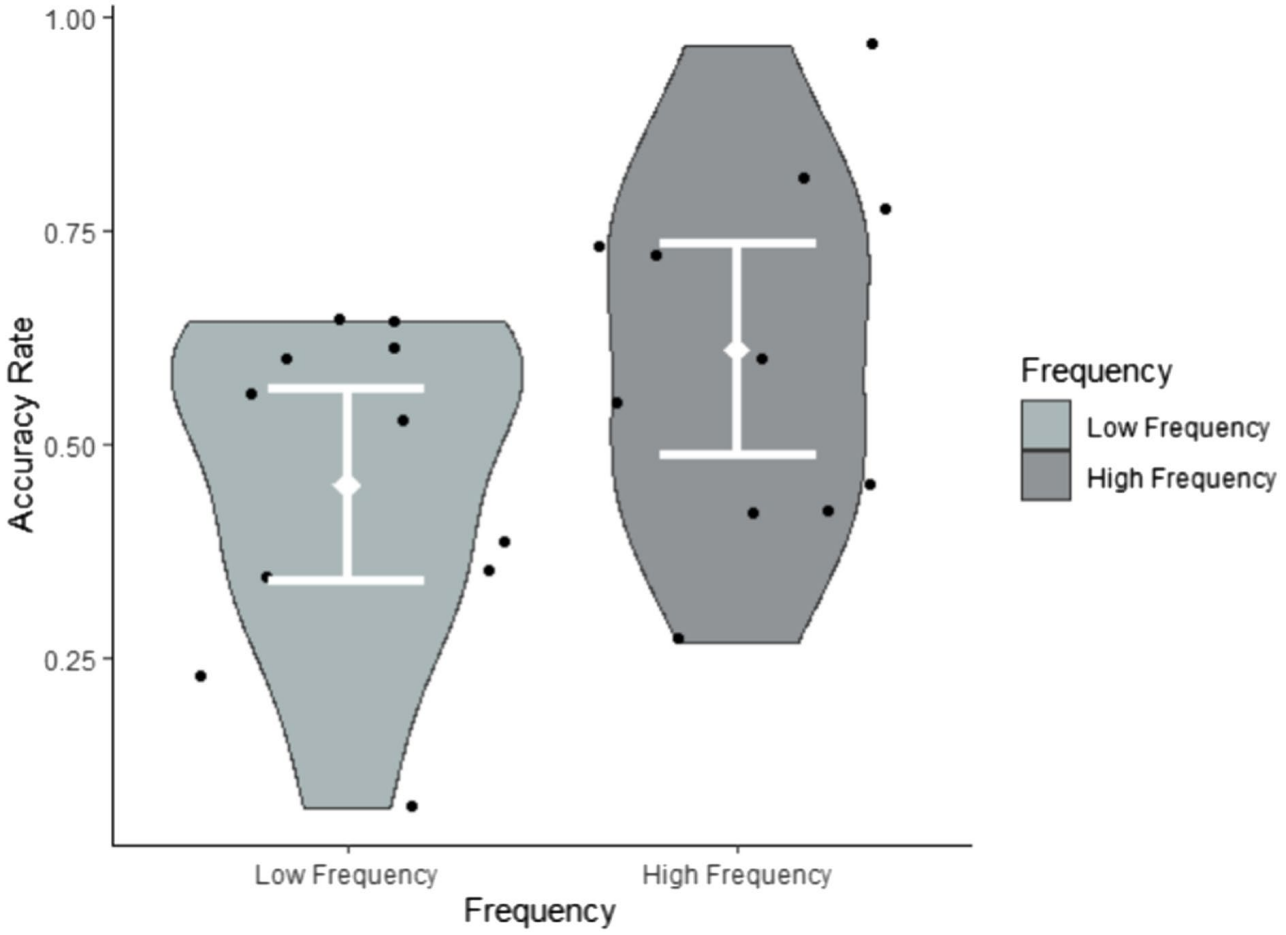
Accuracy rates by frequency summarised by items.

**Figure 6:**
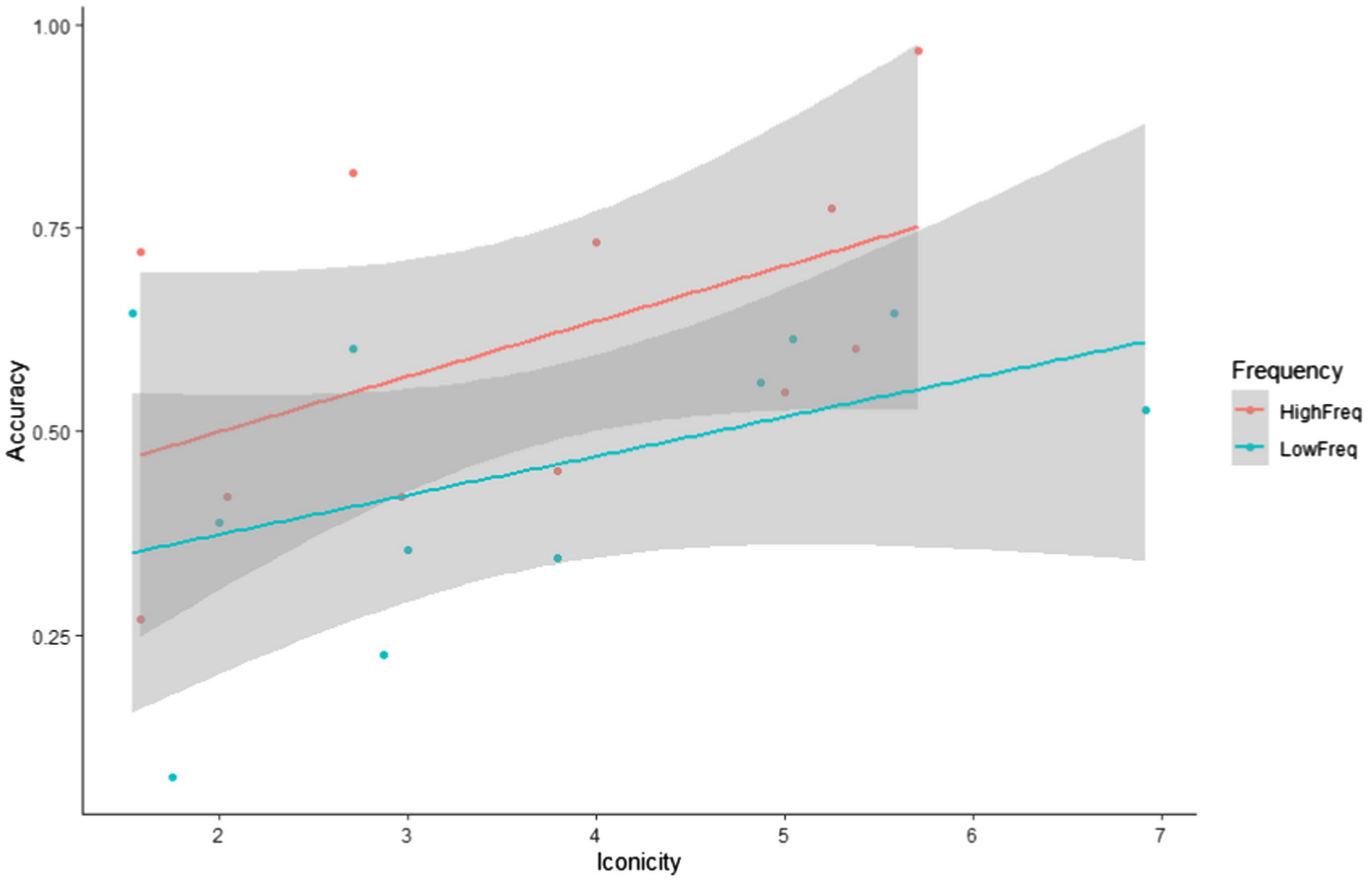
Correlation between iconicity ratings and accuracy rates by frequency.

Research question 2 hypothesised that target sign recognition would be modulated by both the frequency and iconicity of each target item. To explore their impact on target item recognition, we conducted a glmer model (family = ‘binomial’) with Accuracy (accurate, inaccurate) as the dependent variable and Frequency (low, high) and Iconicity (continuous ratings on a scale from 1 = ‘low’ to 7 = ‘high’) as fixed effects. We also added the between-subject factor Exposure group (1x, 2x) to the analysis. Table 6 reveals that the fixed effects of both frequency and iconicity were significant, but that there was no interaction between them. This suggests that frequency and iconicity jointly contributed to recognition in a cumulative fashion, as illustrated in Figures 4–6. Exposure group was not a significant factor and did not interact with the significant fixed effects.

**Table 6:** Model output accuracy by frequency and iconicity.

Random effects	Variance		*SD*	
Subject	0.22		0.47	
Item	0.74		0.86	
Fixed effects	*B*	*SE*	*Z*	*p*
Intercept	0.18	0.20	0.89	0.02
Frequency	0.45	0.19	2.33	0.02
Iconicity	0.50	0.19	2.60	0.001
Exposure group	0.07	0.07	0.95	0.34
Frequency: iconicity	0.11	0.19	0.57	0.57
Frequency: group	0.05	0.05	0.97	0.33
Iconicity: group	−0.01	0.05	−0.16	0.88
Frequency: iconicity: group	−0.01	0.05	−0.20	0.84

Glmer (accuracy ~ frequency*iconicity*group + (1 + 1|subject) + (1 + 1|item), data, family = ‘binomial’).

To further investigate the cumulative effects of frequency and iconicity, as well as possible threshold effects and also to see whether sign recognition relative to chance levels varied as a function of frequency and iconicity, we conducted additional post-hoc analyses. We classified target signs into four categories with four possible frequency–iconicity combinations: (1) items with high frequency and high iconicity, (2) items with high frequency and low iconicity, (3) items with low frequency and high iconicity, and (4) items with low frequency and low iconicity. For the purpose of this grouping, items with iconicity ratings greater than 3.5 were categorised as having ‘high iconicity’, whilst items with iconicity ratings of 3.5 or less were categorised as having ‘low iconicity’. Sign recognition in each of these four frequency–iconicity combinations was then compared to chance. This was achieved with a glmer model by removing the intercept and using the fixed factors in their un-centred format. This analysis revealed that participants only achieved above-chance performance for items that were both highly frequent and highly iconic, that is they only showed clear evidence of recognising items when frequency and iconicity worked in unison. In all other frequency–iconicity combinations, participants performed at chance (see Table 7). This suggests a threshold effect: exposure to an item three times did not boost recognition, but exposure to an item 8 times did. However, this facilitative effect depended on items being highly iconic.

**Table 7:** Model output for accuracy by chance by frequency and iconicity.

Random effects	Variance		*SD*	
Subject	0.23		0.48	
Item	0.83		0.91	
Fixed effects	*B*	*SE*	*Z*	*p*
FrequencyHigh-IconicityHigh	0.98	0.39	2.51	0.01
FrequencyHigh-IconicityLow	0.16	0.42	0.38	0.71
FrequencyLow IconicityHigh	0.16	0.42	0.38	0.71
FrequencyLow IconicityLow	−0.63	0.39	−1.63	0.10

Glmer (accuracy ~ −1 + frequency:iconicity + (1 + 1|subject) + (1 + 1|item), data, family = ‘binomial’).

### The Effects of Individual Differences on Target Item Recognition Accuracy

Research question 3 probed the potential impact of individual differences between participants on target item recognition. We explored demographic background variables, such as Age and Education, as well as cognitive abilities, such as executive functions, verbal skills, and fluid intelligence. The Flanker task and the Visual Search task produced the effects predicted by the experimental paradigm, confirming that the tasks worked and that participants had understood the instructions. The Flanker task resulted in the Flanker effect (incongruent trial RTs > congruent trial RTs): an ANOVA with Congruency (congruent, incongruent) as the within-subject variable showed that RTs in incongruent trials (*M* = 506.41 ms, *SD* = 64.03 ms) were significantly longer than RTs in congruent trials (*M* = 445.53 ms, *SD* = 65.39 ms, *F* = 974.37, *η*^2^ = 0.914, *p* < 0.001). For the visual search task, an ANOVA revealed the expected Visual Search Load effect, that is longer RTs in displays with 15/20 distractors (*M* = 1206.42 ms, SD = 235.12 ms) than in displays with 10/5 distractors (*M* = 993.41 ms, *SD* = 187.74 ms, *F* = 234.16, *η^2^* = 0.718, *p* < 0.001). In addition, we assessed participants’ general language learning aptitude, their vocabulary size in their first language (English) and their specific language background and language learning history. The correlational analyses (available at https://osf.io/ub28n/?view_only=fce4401c7284438d94d1ce52c7879733) did not indicate that individual factors were sufficiently strongly interrelated to justify summarising them into latent variables/principal components. Moreover, the correlational analyses did not reveal any significant relationships between the individual differences factors and target item accuracy, which was in line with the low subject-based variability reported by the above-described glmer models. Hence, we did not explore individual predictors further.

## Discussion

The overwhelming experience when encountering a novel spoken language is of being faced with a seemingly impenetrable continuous stream of speech. Learners of sign languages face a comparable hurdle. Our question was whether sign-naïve adults can extract linguistic information after just a few minutes of exposure to a continuous stream of naturalistic signed input in an implicit learning context, as shown previously for spoken language (Gullberg et al., 2010, 2012). Answering this question is an important step towards elucidating those features and skills that are common to all language learning, regardless of modality, and those that are particularly relevant to learning sign languages.

We created a weather forecast in Swedish Sign Language (STS) and hypothesised that sign-naïve participants would be able to distinguish between signs that they had and had not seen in this input when tested immediately afterwards. We found some evidence of this ability. Participants could correctly reject distractors, particularly the implausible distractors, at above-chance levels, although they did not accept target items at above-chance levels. Nevertheless, accuracy of target sign acceptance was modulated by the properties of the signs, as we discuss in more detail below. Contrary to our prediction, however, participants who had watched the forecast twice did not perform more accurately than those who had seen it only once. It is possible that participants paid less attention to the second showing of the video, especially since they were instructed that they would be viewing the same video twice.

In order to better understand what led to more accurate identification of viewed and non-viewed signs, we explored properties of signs themselves. For target signs, we found that frequency and iconicity both impacted on accurate recognition and indeed had a cumulative facilitative effect on target item recognition. Importantly, participants showed clear evidence of above-chance recognition of items that were both highly frequent in the input and highly iconic. The frequency effect matches what has been found for spoken language learning (Ellis, 2012), including in implicit learning contexts (Gullberg et al., 2010, 2012). The effect of iconicity suggests that participants were better at recognising linguistic forms linkable *via* perceptuo-motor analogy to their existing conceptual representations. This in turn suggests that participants were endeavouring to construct meaning as they viewed the forecast, even though meaning *per se* was not tested by the task.

Our findings contribute to a growing body of research indicating that iconicity supports language learning, regardless of modality (Dingemanse et al., 2015; Ortega, 2017). However, given the visual nature of sign languages, iconicity is likely to be particularly salient for learners of sign languages: the visual scope of much of what we communicate about, coupled with the visual nature of the sign modality, means there are many possibilities for direct iconic mappings between form (hand configuration, movement, and location) and meaning (Perniss et al., 2010). The observed effects of iconicity could be investigated further by drawing upon the distinction between the notions of *iconicity* and *transparency* (Sevcikova Sehyr and Emmorey, 2019). Iconicity describes a recognisable similarity between a sign and its meaning when participants are provided with both the sign and its meaning. Transparency refers to signs to which the correct meaning can be unambiguously assigned without explicitly being given the meaning. It is likely that the signs on which participants performed above-chance level in this study would also be classified as highly transparent. Future research on incidental sign language learning should go into further detail on this matter because transparency might be particularly relevant for meaning assignment in implicit learning contexts.

We predicted that differential performance on phonologically plausible and implausible distractor items would provide insights into how much phonological information about STS participants had extracted. The data indicated that participants were more accurate at correctly rejecting implausible signs than at correctly rejecting plausible signs, suggesting that they recognised some of the phonological properties that are not part of STS. Two possible explanations can be postulated: first, participants actually built some knowledge of STS phonology during the brief exposure, as learners have been shown to do at first implicit exposure of spoken language (e.g., Ristin-Kaufmann and Gullberg, 2014); second, participants drew on their knowledge of gestural movements and related motor schemas in their assessment of what constitutes plausible manual signs, a knowledge that may go beyond just the particular sign language (STS) viewed in our study. Support for this latter view comes from studies showing that gestures can serve as a substrate for sign language learning (e.g., Marshall and Morgan, 2015; Boers-Visker, 2021). Hence, the differences between phonologically plausible and implausible items might have arisen from sensitivity to articulatory ease (from knowledge of either human biomechanics or gesture), rather than from extracting phonological information from the input.

Finally, we predicted that the accuracy with which participants recognised target signs would be modulated by individual differences in their cognitive skills and existing knowledge of spoken languages. Surprisingly, we found no support for this prediction. However, given that mean performance was at chance for some target items, we acknowledge that only limited observations can be made about the correlation between these factors and actual learning. Nevertheless, the absence of correlations between individual differences and performance accuracy raises the question whether implicit learning in first-exposure contexts is modulated by the individual-level factors we assessed. In explicit sign learning studies, there is mixed evidence for an influence of individual language and cognitive differences on initial learning (Williams et al., 2017; Martinez and Singleton, 2018, 2019). Meanwhile, there is considerable debate over the role of individual differences in implicit spoken language learning (Williams, 2009). An important question remains as to when in the learning trajectory, and under what conditions, the individual’s cognitive and linguistic makeup starts to matter.

This preliminary investigation into sign language learning at first exposure opens many avenues for further research. Importantly, we had no post-test to assess whether the recognition effect translated into a longer-term memorisation of sign forms, which is clearly an important step in lexical learning. Furthermore, the effect of iconicity on sign recognition suggests that participants may have engaged in some form of meaning assignment, although the task itself did not test this. Future research should investigate whether sign-naïve participants, in such an implicit learning context, make links between sign forms and their meanings, similar to spoken language findings of Gullberg et al. (2010). Meanwhile, our participants’ relative success at identifying the phonologically implausible distractor signs as not having been present in the forecast suggests that learners might extract information about the phonological properties of the target sign language at first exposure. This should be explored further, potentially by adapting the lexical decision task of Gullberg et al. (2010). Finally, for practical reasons we studied the learning of just one sign language (i.e., STS), by native speakers of the same language (i.e., English), with just one set of input materials. Our study therefore needs replicating in different sign languages, in adults with different spoken languages and with input materials other than a weather forecast, in order to determine the extent to which our findings hold across languages, populations, and contexts.

In conclusion, our results suggest that during only 4 min of naturalistic continuous language input in a new modality, the adult language learning mechanism can extract information about linguistic forms. Adults can detect individual signs in a continuous sign-stream, create memory traces for (some of) them and extract information about phonology. Crucially, input properties may matter more for implicit learning at this initial stage than learner characteristics. Moreover, we observed both modality-general and modality-relevant effects: the adult mechanism for language learning operates similarly on signed and spoken languages as regards frequency, but also exploits modality-salient properties, such as iconicity for signed languages. Our data suggest that despite the considerable learning challenges, adults have powerful learning mechanisms that enable them to make that first important break into a language—even when visual—to recognise word forms and glean linguistic information from unfamiliar linguistic input.

## Data Availability Statement

The datasets presented in this study can be found in online repositories. The names of the repository/repositories and accession number(s) can be found at: https://osf.io/ub28n/?view_only=fce4401c7284438d94d1ce52c7879733.

## Ethics Statement

The studies involving human participants were reviewed and approved by Institute of Education Staff Research Ethics Committee, University College London, London, United Kingdom. The patients/participants provided their written informed consent to participate in this study.

## Author Contributions

JH, LA, VJ, MG, and CM contributed to the design of the study and provided critical revisions. JH, LA, and CM collected the data. JH organised the database and conducted the statistical analyses. JH, MG, and CM wrote the first draft of the paper. All authors contributed to the article and approved the submitted version.

## Funding

This work was funded by an International Academic Fellowship (IAF2016023 awarded to CM) and a Research Grant (RPG-2018-333 awarded to CM, MG, and VJ) from the Leverhulme Trust.

## Conflict of Interest

The authors declare that the research was conducted in the absence of any commercial or financial relationships that could be construed as a potential conflict of interest.

## Publisher’s Note

All claims expressed in this article are solely those of the authors and do not necessarily represent those of their affiliated organizations, or those of the publisher, the editors and the reviewers. Any product that may be evaluated in this article, or claim that may be made by its manufacturer, is not guaranteed or endorsed by the publisher.

## Footnotes

^1^ https://osf.io/ub28n/?view_only=fce4401c7284438d94d1ce52c7879733

^2^ https://osf.io/zsrh7/?view_only=fce4401c7284438d94d1ce52c7879733
